# Facilitators and Barriers to Adherence and Engagement in a Lifestyle Intervention for High-Risk Older Adults: A Multi-Trial Analysis

**DOI:** 10.3390/nu18050747

**Published:** 2026-02-26

**Authors:** Julia L. Sheffler, Zhuo Meng, Trinity Sims, Viviana Gutierrez Caimary, Ravinder Nagpal, Giovanna Mompremier

**Affiliations:** 1 College of Medicine, Florida State University, Tallahassee, FL 32306, USA; 2 College of Nursing, Florida State University, Tallahassee, FL 32306, USA; 3 Anne’s College, Florida State University, Tallahassee, FL 32306, USA

**Keywords:** interventions, nutrition, adherence, lifestyle, dementia

## Abstract

**Background**: High adherence and engagement are critical components of successful lifestyle interventions for health outcomes. Gaps persist in understanding the multifaceted factors influencing older participants’ ability to adhere and engage with health behavior interventions. **Methods**: Using data from two pilot randomized clinical trials evaluating a nutrition program in older adults at increased risk for dementia, we evaluated participant-level factors influencing adherence and engagement with the recommended diet and program. Additional qualitative themes were used to provide context. Both trials are registered on ClinicalTrials.gov (Pilot A: NCT0417176 on 23 March 2021 and Pilot B: NCT06121986 on 30 October 2023). **Results**: Better income, cognitive functioning, emotion regulation, quality of life, and independence in activities of daily living were associated with better dietary adherence, while being non-White, living in a rural area, having higher depressive symptoms, worse health symptoms, and worse sleep quality were negatively associated with adherence. Higher education, cognitive function, anxiety, and previous weight challenges were associated with better program engagement. **Conclusions**: Adherence and engagement were impacted by a combination of individual factors, including cognition, mood, physical health, as well as the broader socioeconomic context. Our findings highlight the ways psychological and social determinants of health may impact adherence to lifestyle interventions.

## 1. Introduction

Suboptimal adherence to lifestyle interventions results in reduced therapeutic efficacy, diminished quality of life, unfavorable health outcomes, and escalated healthcare expenditures [1]. While ensuring adherence and engagement to interventions is challenging [2], evidence suggests that enhancing adherence and engagement to treatment regimens and healthcare recommendations can significantly contribute to better health outcomes, especially for individuals with chronic health conditions [3]. Despite this growing body of work, much of the existing literature focuses on intervention efficacy rather than the contextual factors that influence long-term engagement, leaving important gaps in understanding why adherence varies across individuals and populations.

Adherence and engagement are not shaped by individual choice alone. Instead, they are influenced by broader social, economic, educational, environmental, and healthcare-related conditions that affect an individual’s capacity to initiate and sustain lifestyle change. These conditions align with the social determinants of health (SDOH), where the circumstances in which individuals are born, live, learn, work, and age influence health behaviors, functioning, and quality-of-life outcomes. SDOH are commonly organized into five domains: Economic Stability; Education Access and Quality; Healthcare Access and Quality; Neighborhood and Built Environment; and Social and Community Context [4]. Although this framework has been widely applied to explain disparities in health outcomes, its application to adherence and engagement in lifestyle intervention, particularly among older adults, remains limited.

Later life represents a period in which multiple social determinants of health intersect and intensify, shaping engagement with health-promoting behaviors. Older adults represent a population for which lifestyle interventions are both highly relevant and pose unique challenges. Aging is often accompanied by increased rates of chronic disease [5], physical and functional limitations [6], and changing roles in society [7], all of which may interact or influence engagement in behavioral interventions. However, many studies examining adherence combine age groups, or treat older adults as a broad category, which can limit insight into how age-related social and contextual factors shape adherence and engagement. As a result, there is a need for research that explicitly examines adherence and engagement within older adult populations through a framework that accounts for late-life changes in social determinants.

Within the SDOH framework, education access and quality play a central role in shaping health literacy, self-management, and engagement with lifestyle interventions. Individual differences and personal characteristics may help to characterize which older adults are most responsive to lifestyle interventions by reflecting underlying social and structural conditions that shape health behaviors. Examining this relationship can offer valuable insights for refining and customizing interventions to improve acceptability, scalability, and efficacy. Educational attainment, a key indicator of education access and quality, has consistently been associated with greater adherence to various intervention modifications and treatments [8,9]. However, these factors are often examined independently, rather than as interconnected determinants embedded within broader social contexts.

Social and community context, including social positioning and lived experience, further shapes adherence to lifestyle interventions. The relationship between age and adherence to lifestyle interventions remains inconsistent, with some studies suggesting greater adherence among older adults [10], while others report higher adherence among younger age groups [8,11]. Racial disparities in adherence and retention to physical activity and diet interventions have also been identified [12], with some researchers finding poorer adherence amongst African Americans compared to white individuals [13]. Additionally, adherence rates in lifestyle intervention programs are often lower among women compared to men [14,15]. Together, these findings highlight the importance of considering broader social and structural context when evaluating adherence.

While this study is grounded in the SDOH framework, individual-level psychological factors also play an important role in shaping adherence and engagement. Self-efficacy [16], motivation [16], stress [17], quality of life [18], and cognitive functioning [19] play important roles in sustaining behavioral change. For example, the Self-Efficacy Scale for Adherence to the Mediterranean Diet study found that adherence increased when participants reported greater confidence in their ability to adhere to the diet, alongside positive outcome expectations, autonomous motivation, affective balance, and life satisfaction [20]. Similarly, within a Lifestyle and Cardiovascular Risk Modification program, high perceived self-efficacy regarding eating habits emerged as a significant predictor of adherence [10]. Successful intervention outcomes have also been associated with higher levels of autonomous motivation, self-efficacy, and self-regulation skills [21], while lower perceived stress and higher quality of life have been linked to greater engagement in lifestyle modifications [22,23]. However, their role within the broader SDOH framework in later life remains unexamined.

Social relationships and daily environments shape participation in lifestyle interventions [24]. Factors such as social well-being, relationship status, living situation, and occupational status all play a role in an individual’s overall health profile [25] and capacity to engage in lifestyle interventions. Among older adults, adherence to dietary interventions has been shown to be lower among both employed and unemployed individuals compared to retired counterparts [11]. Conversely, individuals who report greater emotional support, such as close friendships, demonstrate higher adherence to lifestyle intervention programs [26]. Older adults who live with family members or in structured care environments have been found to exhibit healthier dietary patterns [27]. Even so, social context remains insufficiently integrated into models of adherence among older adults.

Physical health constraints and functional capacity intersect with neighborhood and built environment and healthcare access and quality. Health symptoms (e.g., fatigue, pain) and functional limitations can hinder participation in dietary and physical activity components and increase attrition. Among individuals with chronic conditions, fatigue, poor fitness [28], and joint pain [29] are commonly cited barriers to maintaining exercise and diet routines. In contrast, greater independence in activities of daily living and higher baseline physical activity levels are associated with improved adherence and greater likelihood of achieving intervention goals [28,30,31]. Poor sleep quality may further reduce motivation and adherence [32]. These findings highlight the role of functional capacity in shaping adherence within lifestyle interventions.

This study aims to examine the extent to which social, psychological, and functional factors relate to adherence and engagement across two samples derived from pilot clinical trials of a multi-domain nutrition program for older adults. Guided by the SDOH framework, participant characteristics were conceptualized across five domains: education access and quality were represented by educational attainment; economic stability by annual household income and employment status; healthcare access and quality by psychological and cognitive determinants of health-related behavior, including cognitive function, memory complaints, perceived stress, self-efficacy, quality of life, and history of eating disorder symptoms; social and community context by age, sex assigned at birth, race, marital status, living situation, and social support; and neighborhood and built environment by rurality, physical activity level, functional status, ADLs, general symptom burden, pain, sleep quality, and prior weight challenges.

This approach intentionally centers SDOH and psychological factors as fundamental drivers of adherence and engagement in lifestyle interventions, emphasizing that sustained dietary change is shaped not only by individual effort, but by the social, economic, and healthcare conditions in which people age. Engagement with dietary interventions is therefore examined through the combined influence of cognitive and motivational capacity, emotional and stress-related experiences, and self-regulatory processes, all of which unfold within broader social and structural contexts. By integrating these measured psychological, social, and functional factors across key SDOH domains, this study seeks to explore how later-life social conditions and health-related constraints may shape older adults’ ability to engage with, sustain, and benefit from dietary and behavioral interventions.

## 2. Materials and Methods

### 2.1. Participants

Data was drawn from two pilot randomized clinical trials, Pilot A and Pilot B, which examined two iterations of the same nutrition and lifestyle program designed for older adults at risk for Alzheimer’s disease and other dementias. Both trials were preregistered on ClinicalTrials.gov (Pilot A: NCT0417176 on 23 March 2021 and Pilot B: NCT06121986 on 30 October 2023). These pilot trials were selected for the current study in order to better characterize factors that relate to nutrition adherence across slightly different environments, as well as to better understand how changes in the program may have influenced participant adherence factors. Participants aged 60–85 years in Pilot A and aged 55–85 years in Pilot B with possible mild cognitive impairment (MCI) or normal cognition living in the community were recruited. Randomization was completed using the National Institutes of Health clinical trial randomization tool using a maximum tolerated imbalance of 3 to maintain approximately equal participants per arm as recruitment progressed. Using this pre-generated randomization list, participants were allocated to an arm in order of their enrollment in the study. Study investigators completing analyses were blinded to the participant arm allocation to reduce potential bias. Inclusion criteria included age, interest in nutrition program, internet access, English proficiency, and Memory Complaint Score (MCS) above 1, converted Montreal Cognitive Assessment (MoCA) total score: MCI = 17 < 26, CN + >26 and/or MCS MCI > 3, CN < 3, stable medical condition (at least 3 months prior to screening visit), and stable on medications at least 4 weeks prior to screening visit. Participants were excluded for major medical or psychiatric conditions, as well as a nut or fish allergy, current use of any restrictive or specialized diet, a body mass index (BMI) less than 19 kg/m^2^, non-English speaking, intake of potentially conflicting medications (including insulin, monoamine oxidase inhibitors [MAOIs], or immunosuppressive drugs, Warfarin, or any other drugs with potential central nervous system effects or anticholinergic activity), or cognitive impairment too advanced for study participation (i.e., MoCA < 19 in Pilot A and MoCA ≤ 16 in Pilot B). Thus, participants included both older adults who were cognitively normal or had possible MCI.

### 2.2. Pilot Trial Design

Pilot A (*N* = 58) was a two-arm, block randomized pilot clinical trial investigating the use of motivational interviewing (MI) and cognitive behavioral therapy (CBT) techniques to enhance adherence to a Modified Mediterranean ketogenic diet (MMKD) through the Improving Cognitive Aging through Nutrition (ICAN) program. One arm contained an education-only component (*N* = 29), whereas the other incorporated MI-CBT techniques embedded within the program’s sessions (*N* = 29). Therefore, this pilot aimed to evaluate whether MI-CBT strategies enhanced adherence to an MMDK within a group setting, compared to the group with an education-only component. A detailed description of Pilot A methods, the tointervention, and primary outcomes have been previously reported [33].

Pilot B (*N* = 65) expanded on the findings of Pilot A using a 2 × 2 factorial design in which participants were assigned to one of four study arms: (1) MMKD ICAN program + ongoing support group (*N* = 16), (2) MMKD ICAN program (*N* = 17), (3) Mediterranean diet (MED) ICAN program + ongoing support group (*N* = 15), or (4) MED ICAN program (*N* = 17). Based on the results demonstrating that the MI-CBT arm was associated with better adherence, retention, and engagement from Pilot A [33], all arms included the MI-CBT components. Of note, 3 of the 65 participants were assigned to a group non-randomly (due to factors unrelated to study arm, e.g., group time/day availability), and one participant was withdrawn from the study prior to beginning the program due to a dementia diagnosis not identified at screening. It is also important to emphasize that the current analyses did not explore differences between the four arms, as the primary focus is on non-program factors affecting adherence to the program more broadly.

For both pilots, comprehensive clinical and psychosocial data was collected through in-person assessments completed at baseline (within 2 weeks of starting the program) and post-intervention (within 2 weeks of ending the program). Additionally, during the intervention period, participants were e-mailed short weekly surveys to collect additional information about dietary adherence (e.g., daily urine ketones, self-rated adherence, MEDAS) and psychosocial factors that might influence their participation or safety (e.g., health symptoms, mood, stress). Measures were selected to capture participant characteristics previously associated with adherence to lifestyle interventions in older adults. These included demographic, clinical, and psychosocial variables that may influence an individual’s capacity to initiate and sustain dietary change. Selection was informed by prior research on behavioral intervention engagement and by feasibility considerations within a community-based pilot trial. For the current analyses, quantitative data was analyzed from baseline and post-intervention (6 weeks for pilot A and 10 weeks for Pilot B). Data for both pilot studies was stored using Research Electronic Data Capture (REDCap v15.5.23). Qualitative data was collected via semi-structured exit interviews conducted by trained research assistants at post-intervention assessments. Interviews were recorded, transcribed, and reviewed by at least two researchers. In the present study, a hybrid coding methodology, integrating both a-priori and emergent codes to identify common themes, was used to analyze transcripts and provide context for quantitative data.

### 2.3. Intervention Design

The ICAN program, used in both pilots, consisted of one-hour sessions, except for the first session, which lasted 90 min. Sessions were delivered via HIPAA-compliant Zoom with the first session being held in person in Pilot B. Nutrition information and psychoeducation components were presented using pre-recorded video slides. During sessions, participants were provided with detailed guidance on identifying, purchasing, and planning meals based on each dietary plan, and participants had full autonomy for purchasing and preparing their own meals. All participants received a workbook containing handouts aligning with the weekly session content (e.g., nutrition, goal setting, macronutrient tracking) and a cookbook. Weekly online surveys were collected throughout the intervention sessions. This intervention package was developed using the Information-Motivation-Behavioral skills model [34]. Sessions were led by two trained facilitators who guided participants through educational content, structured skills practice, and opportunities for discussion and questions. Facilitator training included passing nutrition knowledge checks, reviewing all program manuals and presentations, completing an MI-CBT workshop, and completing additional relevant readings and peer-practice sessions. To maintain intervention fidelity, all sessions were recorded, reviewed, and rated by a licensed psychologist and certified nutrition coach using the ICAN fidelity checklist. This checklist was designed to evaluate the consistent use of MI and CBT skills, accurate nutrition guidance, as well as appropriate use of session prompts and materials. Facilitators were supervised by a licensed psychologist and registered dietitian.

### 2.4. Measures

#### 2.4.1. Sociodemographic Factors

Age, sex assigned at birth (0 = female, 1 = male), race (0 = White, 1 = Black/other), and education (0 = less than a bachelor’s degree, 1 = bachelor’s degree, or 2 = more advanced degree) were included in the analyses for both Pilot A and Pilot B. Annual household income (0 = less than $50,000 vs. 1 = $50,000 or more) and rurality (0 = no vs. 1 = yes) were available for Pilot B only.

#### 2.4.2. Psychological Factors

Cognitive Function was assessed using the Telephone Montreal Cognitive Assessment (T-MoCA), with scores converted to the MoCA-30 scale using the equipercentile equating method [35]. Scores of 26 or above were considered normal, whereas scores between 18 and 25 suggest possible MCI [36].

Memory Complaints were measured by the 7-item Memory Complain Scale (MCS) [37]. Each item was rated on a three-point Likert scale (0–2), yielding a total score ranging from 0 to 14 (higher indicates worse memory concerns).

Depression was evaluated using the 9-item Patient Health Questionnaire (PHQ-9) [38,39]. Participants rated the frequency of depressive symptoms experienced over the past two weeks on a four-point scale ranging from 0 (“not at all”) to 3 (“nearly every day”). Total scores range from 0 to 27.

Anxiety. The 10-item Geriatric Anxiety Scale (GAS-10) was utilized to assess anxiety symptoms among older adults [40,41]. Each item was rated on a four-point scale ranging from 0 (“Not at all”) to 3 (“All of the time”), based on the frequency of symptoms experienced over the past week. Total scores range from 0 to 30.

Stress. The 10-item Perceived Stress Scale (PSS-10) [42,43] assessed stress during the last month using a 5-point Likert scale from 0 (“never”) to 4 (“very often”). Four positively worded items (items 4, 5, 7, and 8) were reverse coded so that higher scores indicate higher levels of perceived stress. Total scores range from 0 to 40.

Emotion Regulation. The Positive and Negative Affect Schedule (PANAS) [44,45] was employed in Pilot A, which consists of 20 items measuring positive and negative feelings over the past week. Sum scores ranging from 10 to 50 were calculated separately for positive and negative affect. In Pilot B, participants’ emotion regulation strategies were assessed using the 10-item Emotion Regulation Questionnaire [46,47]. Items were rated on a 7-point scale from strongly disagree (1) to strongly agree (7). Two subscale scores were computed: cognitive reappraisal (six items) and expressive suppression (four items).

Self-Efficacy was evaluated using the 10-item Self-Efficacy (GSE) Scale [48,49]. Participants rated how true each statement was for them on a four-point scale from not at all true (1) to exactly true (4).

Previous Eating Disorder was assessed using the SCOFF questionnaire [50,51], a five-item yes/no screening tool designed to identify individuals at risk for eating disorders. Higher total counts suggest an increased likelihood of having an eating disorder.

Quality of Life was measured using the 13-item Older People’s Quality of Life questionnaire (OPQOL) [52], range = 13 (worst)–65 (best). Responses were recorded on a 5-point Likert scale from 1 (“strongly disagree”) to 5 (“strongly agree”).

#### 2.4.3. Social and Behavioral Factors

Living Situation. Participants were asked to report on whom they were living with. The responses were dichotomized into living alone or living (0) with at least one other person (1).

Occupation Status. Employment and retirement status were evaluated. Participants were grouped into two categories: not currently working (0) and currently working (1).

Interpersonal Support was assessed using the 12-item Interpersonal Support Evaluation List (ISEL), which evaluates perceptions of social support including appraisal support, belonging support, and tangible support [53,54]. Each item was rated on a 4-point scale ranging from 1 (“definitely false”) to 4 (“definitely true”). Six negatively worded items were reverse coded, and a total sum score was calculated.

Physical Activity. Participants reported their physical activity levels by selecting one of five categories from little or no exercise to very hard exercise/sports or a physically demanding job. Responses were dichotomized into inactive (0) and active groups (1), with “active” defined as engaging in moderate exercise or more.

#### 2.4.4. Medical and Functional Factors

Functional Status. In Pilot A, the Functional Status Questionnaire (FSQ) [55] was utilized to evaluate participants’ physical, psychological, social, and role functioning during the past month, and was divided into six subscales. Average scores for each subscale were calculated based on valid items and linearly transformed to a 0–100 scale, with higher scaled scores representing better functioning.

General Symptoms. In Pilot B, participants reported how often they were bothered by a list of 30 general symptoms in the past 2 weeks on a five-point scale from 0 = “not at all” to 4 = “very much” [56]. A sum score ranging from 0 to 120 was calculated.

Previous weight challenge. In Pilot A, participants reported whether they had experienced struggles with weight.

Pain was measured by 24 items adapted from the Roland–Morris Disability Questionnaire (RMDQ) assessing functional disability related to lower back pain [57]. In our studies, we expanded the items to general pain. Participants indicate whether each statement applies to them (“Yes” = 1, “No” = 0), with a total score range of 0 (no pain) to 24 (severe pain).

Sleep Quality. The Pittsburgh Sleep Quality Index (PSQI) [58] was used to assess participants’ sleep quality. Seven component scores including subjective sleep quality, sleep latency, sleep duration, sleep efficiency, sleep disturbance, use of sleep medication, and daytime dysfunction were calculated based on participants’ corresponding survey responses, with each component ranging from 0 to 3. A global sum score ranging from 0 to 21 was calculated, with higher scores indicating poorer overall sleep quality.

### 2.5. Outcome Measures

#### 2.5.1. Adherence

Ketone Adherence. Participants measured daily morning acetoacetic acid levels using ketone test strips and recorded the date and corresponding values throughout the intervention period. Ketone adherence was defined as the percentage of recorded days with ketone levels above the trace threshold. Participants with recordings for fewer than two-thirds of days were treated as missing. Note that in Pilot B, ketone levels were measured only for participants assigned to the MMKD arms (arms 1 and 2).

Perceived Adherence. Participant adherence to the nutrition program was evaluated via self-report on a 0 (not at all)–10 (All the time, very consistent) scale. Self-rated adherence was collected weekly through online surveys administered throughout the intervention periods for both Pilot studies. An overall perceived adherence score was calculated by averaging all self-reported adherence ratings across the follow-up period, with participants providing fewer than two-thirds of ratings considered missing.

Mediterranean Diet Adherence. Adherence to the MED was measured using the 14-item Mediterranean Diet Adherence Screener (MEDAS) [59] in Pilot B. Each item was scored 0 or 1, based on whether participants met specific dietary criteria. Total scores range from 0 to 14.

#### 2.5.2. Engagement

Session Attendance. Participants’ engagement with the intervention was assessed as the percentage of weekly sessions attended throughout the intervention period in both pilot studies.

Submission of Food Logs. Participants were required to submit food logs, either handwritten or electronic, at least three times per week throughout the intervention period. A binary variable was created to indicate whether participants submitted food logs.

### 2.6. Analyses

#### 2.6.1. Quantitative Analysis

Quantitative analyses were conducted using R v4.5.0. First, sample characteristics for both pilot studies were summarized using descriptive statistics. Differences in participant characteristics between the two studies were assessed using *t*-tests for continuous variables and chi-square tests for categorical variables. Accounting for differences between the studies, correlations between each baseline factor and each outcome measure were then evaluated separately within each pilot study. Pearson or Spearman correlations (*r*) or the *ϕ* coefficient which ranges from −1 to 1 were calculated depending on variable type and distribution. Normality of each continuous variable was assessed using the Shapiro–Wilk test. The significance level was set at the conventional threshold of *α* = 0.05. To mitigate the effects of small sample size and variable imbalance, 95% bootstrap confidence intervals (CIs) for all correlations were estimated and reported using 1000 replicates. As these analyses were intended as exploratory and descriptive, we prioritized reporting effect sizes and 95% bootstrap CIs rather than significance. To aid transparency and controlling for multiple testing, *p*-values and false discovery rate (FDR)-adjusted *p*-values were reported in the tables as supplemental information. Given the differences in study design and measures employed between Pilot A and Pilot B, the two pilots were analyzed and reported separately.

#### 2.6.2. Qualitative Analysis

Audio recordings from each study interview were transcribed and subjected to reflexive thematic analysis, as described by Braun and Clarke [60]. The analysis followed Braun and Clarke’s six-phase approach: familiarization with the data, generating initial codes, searching for themes, reviewing themes, defining and naming themes, and producing the report. An inductive, data-driven approach was used to allow themes to emerge directly from the transcripts rather than being guided by pre-existing theoretical framework.

Two coders independently conducted an initial review of all transcripts. Each coder generated preliminary codes and identified patterns related to facilitators and barriers. Following independent coding, the coders met to compare coding structures, discuss discrepancies, and refine interpretations to enhance analytic consistency. Disagreements that could not be resolved through discussion were reviewed by a third investigator to reach final consensus. Coding, organization, and interpretative analysis were facilitated by NVivo 20 software [61].

To further strengthen methodological rigor, themes were systematically reviewed and refined by the research team to ensure they accurately represented and were grounded in the data. Qualitative findings were examined alongside quantitative survey results to provide a more comprehensive understanding of facilitators and barriers. Final themes were agreed upon by both independent coders to enhance consistency and reduce potential bias in interpretation. All available transcripts were included in the analysis, and themes were refined throughout the analytic process. As analysis progressed, core themes were established during earlier stages of coding, and subsequent interviews largely reinforced these patterns rather than introducing substantially new thematic categories, suggesting adequate thematic depth within the dataset.

## 3. Results

### 3.1. Sample Characteristics

Table 1 summarizes the sample characteristics for Pilot A (*N* = 58) and Pilot B (*N* = 65). Notably, significant differences were observed in cognitive function, memory complaints, physical activity, group cohesion, and all outcome measures. Compared with participants in Pilot A, those in Pilot B exhibited greater cognitive impairment, more memory complaints, and lower levels of physical activity at baseline. With respect to outcome measures, participants in Pilot B demonstrated lower diet adherence, but higher engagement in terms of session attendance and food log submission.

### 3.2. Economic Stability

Complete results are shown in Appendix A Table A1, Table A2, Table A3, Table A4 and Table A5. Economic stability factors, including household income and employment status were associated with adherence and engagement, particularly in Pilot B. Household income was positively correlated with both session attendance (*r* = 0.26, 95% CI [0.02, 0.49]) and food log submission (*ϕ* = 0.28, 95% CI [0.04, 0.52]).

Qualitatively, participants with higher income or the ability to retire described fewer barriers related to transportation, food affordability, and scheduling flexibility for program participation. Several mentioned that financial stability or flexible work arrangements allowed them to better manage time for meal preparation and logging activities. For example, one participant stated, “… *I’m glad I’m retired. I had all the time I needed. It was time-consuming*” when asked about food logging. In contrast, participants with lower or fixed incomes described more obstacles to maintaining consistency, often citing the higher cost of healthy foods (e.g., fruits, vegetables, nuts), limited time, or competing responsibilities. For example, one participant stated, “*this way of eating could be a lot more expensive*… *because the junk food is a lot cheaper, and it’s a lot easier, and you don’t have to plan*” and another described work obstacles, “… *I’m still working, and it’s really hard when you go to a conference, and the only food they give you is what’s on the plate in front of you*… *That was tough.*” These descriptions reflect differences in time availability and financial flexibility among participants, consistent with the association observed in the quantitative data.

### 3.3. Education Access and Quality

Educational attainment was associated with engagement behaviors. Participants with higher levels of education were more likely to submit food logs (*ϕ* = 0.26, 95% CI [0.02, 0.50]), suggesting that educational resources and health literacy may support adherence-related behaviors. These findings were observed across pilots and reinforce the possible role of education as a structural determinant related to engagement rather than an isolated individual characteristic.

### 3.4. Social and Community Context

Social and community context factors, including race, sex, living situation, interpersonal support, and group dynamics, were associated with adherence and engagement outcomes. In Pilot A, race was negatively correlated with ketone adherence (*r* = −0.45, 95% CI [−0.70, −0.22]) and perceived adherence (*r* = −0.38, 95% CI [−0.68, −0.10]), indicating Black participants had lower ketone levels and perceived adherence. However, this finding should be interpreted cautiously given the group imbalance, and such associations were not observed in the more racially diverse sample in Pilot B.

In Pilot B, participants living with others demonstrated higher perceived adherence compared to those living alone (*r* = 0.36, 95% CI [0.14, 0.59]), suggesting that living with others may be beneficial for adherence. This pattern was also reflected in the qualitative interviews for both pilots. Participants who reported living with a spouse, family member, or roommate described shared meals and encouragement that supported consistent participation. For example, one participant described her roommate as, “*my cheerleader through this*” and another stated that “*…my wife and I enjoyed… plotting the daily meals… we work together with trying to figure out the gram requirements…*” Those living alone more often reported difficulty staying motivated or preparing full meals. For example, one participant stated, “*I just found it difficult to find and fix those [meals] for one person. If I had somebody else in the house, that might have been a motivating factor.*”

### 3.5. Healthcare Access and Quality

Healthcare access and quality were reflected through psychological and cognitive factors influencing participants’ capacity to engage with and sustain recommended behaviors. Cognitive function was positively associated with ketone adherence (*r* = 0.41, 95% CI [0.08, 0.78]) and perceived adherence (*r* = 0.28, 95% CI [0.03, 0.54]) in Pilot A, as well as session attendance in Pilot B (*r* = 0.33, 95% CI [0.08, 0.56]). However, in Pilot B, baseline cognitive function was negatively associated with ketone adherence (*r* = −0.38, 95% CI [−0.70, −0.07]). Memory complaints were associated with lower likelihood of food log submission in Pilot A (*r* = −0.26, 95% CI [−0.50, −0.01]).

Regarding mental health, higher depression was associated with lower perceived adherence in Pilot B (*r* = −0.36, 95% CI [−0.58, −0.16]), whereas higher anxiety (*r* = 0.29, 95% CI [0.04, 0.54]) and perceived stress (*r* = 0.24, 95% CI [0.01, 0.48]) were linked to greater session attendance in Pilot A. With respect to emotion regulation in Pilot B, greater expressive suppression was associated with higher ketone adherence (*r* = 0.35, 95% CI [0.00, 0.74]), while cognitive reappraisal was correlated with higher MED adherence (*r* = 0.42, 95% CI [0.17, 0.68]) but lower likelihood of food log submission (*r* = −0.27, 95% CI [−0.54, −0.01]). Finally, in both studies, quality of life was positively associated with perceived adherence (Pilot A: *r* = 0.36, 95% CI [0.06, 0.65]; Pilot B: *r* = 0.27, 95% CI [0.03, 0.52]). = 0.27, 95% CI [0.03, 0.52]).

Information collected in qualitative interviews reflected many of these findings. For example, participants describing low motivation, fatigue, or emotional difficulty reported challenges maintaining adherence or completing program activities. For example, one participant stated, “*There’s been some emotional things. I’ve been doing a lot of emotional eating at night. So okay, but since I figured, hey, I blew this keto already might as well blow it completely.*” In both pilot studies, quality of life was positively associated with perceived adherence, and participants frequently described physical and emotional improvements that aligned with higher self-rated adherence. For example, participants stated, “*I feel more energetic after this program… so I will try to stick to the program and see how I’m doing*” and “*I feel good… I don’t feel anything like I thought I was supposed to feel [at this age]*” and “*I think mentally I’m sharper…*” These types of responses corresponded with quantitative associations between mental health indicators and adherence behaviors observed across both pilot studies.

### 3.6. Neighborhood and Built Environment

Neighborhood and built environment factors, reflected by rurality, physical activity, functional status, symptom burden, pain, and sleep quality, were strongly associated with adherence outcomes.

Rurality was negatively correlated with Mediterranean Diet Adherence Screener (MEDAS) scores in Pilot B (*r* = −0.27, 95% CI [−0.52, −0.02]), suggesting lower dietary adherence among participants living in rural areas. This relationship was further supported by qualitative interviews, in which rural participants described logistical and environmental challenges affecting adherence, including limited access to grocery stores, higher food prices, and fewer available ingredients for healthier meals. Commonly mentioned issues included limited access to grocery stores (e.g., distance), higher food prices, and fewer available ingredients for healthier meals.

In Pilot A, better instrumental activities of daily living were correlated with higher ketone adherence (*r* = 0.40, 95% CI [0.18, 0.60]) and perceived adherence (*r* = 0.41, 95% CI [0.22, 0.62]). Further, participants with previous weight challenges were more likely to attend sessions (*r* = 0.31, 95% CI [0.03, 0.59]) and submit food logs (*ϕ* = 0.31, 95% CI [0.01, 0.61]) in Pilot A.

In Pilot B, higher general symptom burden was associated with lower perceived adherence (*r* = −0.41, 95% CI [−0.63, −0.21]) and lower MED adherence (*r* = −0.29, 95% CI [−0.56, −0.02]). Higher levels of pain were also linked to lower MED adherence (*r* = −0.27, 95% CI [−0.55, −0.01]), and poorer sleep quality was associated with lower perceived adherence (*r* = −0.31, 95% CI [−0.55, −0.07]).

Consistent with these findings, in qualitative interviews, many participants related physical activity and energy to adherence. For example, one participant stated, “*It is my stamina … so when I walked before, I was like really tired. Well, now I’m not as tired. You know… I have more stamina.*” Other participants who described feeling physically stronger, experiencing less joint pain, and becoming more mobile also reported greater ease managing meals and maintaining participation in the program. Unfortunately, pain and general symptoms were also reported in qualitative interviews as barriers to adherence. Some participants reported constipation and leg cramps as factors that negatively impacted their decision to stay consistent with the diets. For example, participants stated, “*the fact that I was constipated almost the entire time, just made me not want to follow it*” and “*When I did get into ketosis, I had severe leg cramps*… *I don’t want anything to do with it.*” These findings underscore the critical influence of functional and symptomatic changes on adherence behaviors within metabolic health interventions.

The theme of prior struggles with weight contributing to greater engagement was also reflected in the qualitative themes. Specifically, participants noted that the structure and accountability of the program helped them regain a sense of control around food. Several participants referred to prior weight management experiences or familiarity with structured programs as supportive factors. For example, participants stated, “*I’ve done a lot of different food programs… I’m very interested in nutrition*” and “*I think [the program] was a good impact on my weight, which I’ve fought all my life*… *I felt good.*” For many individuals, the social aspects of the program were particularly helpful. For example, one participant stated, “*the people were very supportive of the questions… the answers were really professional… very helpful.*”

## 4. Discussion

In this multi-trial analysis, we explored the barriers and facilitators of adherence to a nutrition and lifestyle program in high-risk older adults across two pilot studies. Our study offers a novel perspective that incorporates qualitative context about participant adherence dynamics in community lifestyle programs. The findings highlight the complexity of adherence and engagement in lifestyle programs, which can be affected by personal characteristics, health and functional status, psychological and social context, and program components. Further, the analyses, which used data from two studies examining the same program that was modified over time, demonstrated that relatively minor changes to a program’s structure and content can significantly influence the individual-level factors associated with adherence and engagement.

Several sociodemographic factors were associated with dietary and program adherence across the two cohorts. In Pilot A, Black participants reported lower adherence; however, in the more racially diverse sample from Pilot B, no racial differences in adherence were identified. These discrepant findings may suggest that, while adherence may at times be shaped by racial or ethnic background, inclusion of a representative sample may eliminate such biased findings. Similarly, inclusion of a more geographically representative sample in Pilot B more clearly demonstrated the adherence challenges faced by rural participants who tended to describe more challenges related to accessing healthy foods and higher food costs. These findings are consistent with prior research showing that factors affecting adherence to a MED included limited access to healthy foods and cost [62]. Household income was positively associated with engagement behaviors such as attendance and food log submission, and participants with greater financial resources or more flexible schedules (e.g., retirees) described fewer obstacles to meal preparation and self-monitoring. Educational attainment was also associated with more consistent food log submission, which may have implications for measurement of engagement and adherence for future trials. Specifically, individuals who are more academically inclined may be more likely to engage with components of programs that are similar to academic practices (e.g., class attendance, homework completion). Although it was notable that income and education were not associated with dietary adherence.

Overall, these findings point to a few key considerations when designing and testing lifestyle programs for diverse older adults. First, including both geographically and racially diverse samples may be critical to evaluate both the effectiveness of an intervention and identifying barriers to real-world implementation. Further, it may be necessary to address issues of socioeconomic disadvantage and rural access to healthy foods as part of an effective program. Neglecting these issues may lead to frustration and low long-term sustainment among diverse individuals. Interventions may therefore benefit from incorporating cost-conscious meal strategies, greater flexibility in program activities, and support tailored to the needs of individuals facing logistical and financial constraints. Finally, the effects of education and income also point to potential design considerations in structuring programs similar to a traditional educational class structure versus taking alternate approaches to support engagement and adherence.

Psychological and cognitive factors were also associated with variation in dietary adherence and program engagement. Better cognitive function and fewer memory complaints were linked to higher adherence in Pilot A and to higher session attendance in Pilot B. Although there was a negative association between cognitive function and ketone adherence in Pilot B, this may reflect the higher proportion of participants with MCI in that cohort or may align with modifications to the intervention for Pilot B to specifically support memory retention for individuals with MCI. Consistent with previous studies, higher depressive symptoms were related to lower perceived adherence in Pilot B [63]. Meanwhile, higher anxiety and stress were unexpectedly associated with greater session attendance. Given that the average level of anxiety and stress in the sample was relatively low, this finding may point to the potential benefits of eustress or anxiety in motivating engagement for some participants [64]. Patterns in emotion regulation also corresponded with adherence behaviors, with expressive suppression associated with higher ketone adherence and cognitive reappraisal linked to higher MED adherence but lower food log completion.

Qualitative data supported many of these relationships and elucidated additional themes. Participants who described emotional eating, low motivation, or fatigue often reported greater difficulty maintaining dietary consistency. Conversely, improvements in mood, energy, and perceived cognitive sharpness were commonly described by participants who reported higher adherence, aligning with positive associations between quality of life and perceived adherence observed in both studies. Together, these findings highlight the potential role of psychological well-being, cognitive capacity, and emotional coping strategies in shaping adherence and engagement. Lifestyle interventions for older adults may benefit from integrating behavioral support, stress management, and other coping strategies to address emotional and cognitive barriers.

Although there were relatively fewer social factors related to adherence and engagement, one important relationship was evident in both Pilots. Specifically, participants who lived with supportive others showed higher perceived adherence than those living alone. This association was consistently reflected in the qualitative data, where participants described how shared meal preparation and encouragement from spouses, family members, or roommates supported consistency, whereas those living alone often reported difficulty sustaining motivation and preparing meals for one. Prior lifestyle programs have already demonstrated benefits of taking these types of approaches [65]. Together, these results suggest that social support and context likely play significant and meaningful roles in sustaining adherence. Programs may therefore benefit from incorporating partner- or peer-based support strategies, meal planning tools for single-person households, and structured accountability features tailored to participants with varying levels of prior experience with dietary change.

Participants’ health symptoms and physical functioning influenced different aspects of adherence across the two pilots. In Pilot A, participants with better ability to carry out daily activities showed higher overall adherence, and qualitative accounts suggested that increased stamina, mobility, and physical comfort supported participants’ ability to plan meals and maintain engagement. Additionally, participants in Pilot A with previous weight management challenges were more likely to attend sessions and submit food logs, and many described the program’s structure, accountability, and social support as aligning with strategies that had helped them in the past. This finding was similarly echoed in the interviews, where participants frequently cited existing health concerns as a motivating factor influencing their desire and ability to continue using the recommended diets. In contrast, in Pilot B, greater general symptoms, higher levels of pain, and poorer sleep quality were associated with lower dietary adherence. In interviews, participants also described constipation, leg cramps, and pain as barriers that reduced their willingness to continue the dietary protocol. Notably, across both pilots, participants indicated that improved health symptoms, such as improved sleep quality, increased energy, and reduced pain levels contributed to better dietary adherence and plans to maintain the respective diets long-term.

These findings highlight that physical functioning and symptom burden can either facilitate or undermine adherence. Interventions may therefore benefit from careful assessment of existing health conditions and symptoms in order to develop individualized goals for participants to enhance motivation and engagement. Further, monitoring and proactively managing common side effects, integrating guidance for common symptom relief, and tailoring dietary adjustments to support participants with mobility limitations or chronic pain will likely increase sustained engagement.

### Limitations

There are some important limitations to our study that should be acknowledged. First, while Pilot B included a relatively representative sample, the sample from Pilot A was primarily white and from a slightly higher socioeconomic status. This limitation hinders the generalizability of our findings and may overlook important nuances in adherence behaviors across different racial and demographic groups. Additionally, it is important to note that this study is an evaluation of two different pilot studies, with Pilot Study B being an expanded and modified version of Pilot Study A. Some variables included in Pilot Study B were not included in Pilot Study A. Differences in measurement and intervention design may affect the comparability of results between these two studies. Further, both samples included relatively motivated volunteers with interest in changing their diet and who also were required to have internet access. These factors may impact generalizability to broader clinical patient settings, where patients may be referred without intrinsic interest in changing behavior, or may not have access to the digital tools necessary to engage in this type of intervention. Finally, it is important to acknowledge that the small sample sizes in each of the pilots may lead to over- or underestimation of the reported effects, which may also partially account for discrepancies in results across the two trials. Future, fully powered trials should re-examine these effects to determine whether our findings persist in larger and more representative participant populations.

## 5. Conclusions

In summary, our findings underscore the multifactorial nature of adherence and engagement in nutrition and lifestyle programs for older adults. Adherence may be shaped not only by individual factors such as cognitive function, mood, and physical health, but also by social context, economic resources, and program design. Addressing socioeconomic and access-related barriers, tailoring behavioral and cognitive supports, and integrating flexible strategies that accommodate varying functional abilities may enhance long-term engagement and real-world effectiveness. Future research should build on these exploratory insights through adequately powered randomized trials that evaluate the impact of scalable supports, such as ongoing peer or partner-based accountability, cost-conscious meal planning, and adaptive materials for individuals with cognitive or physical limitations. Longitudinal designs that track adherence, health, and cognitive outcomes over extended periods will be critical for understanding how nutrition and lifestyle interventions can better help participants sustain positive behavioral changes and promote healthy aging.

## Figures and Tables

**Table 1:** Sample characteristics.

	Pilot A (*N* = 58)		Pilot B (*N* = 65)		
Measures	*N*	Statistics	*N*	Statistics	Difference
***Sociodemographic Factors***					
Age	58	72.9 ± 6.3	65	70.2 ± 5.6	*
Sex, male	58	11 (19.0%)	65	16 (24.6%)	
Race, non-White	57	8 (14.0%)	62	33 (53.2%)	***
Education, Bachelor’s degree	57	14 (24.6%)	65	17 (26.2%)	
Education, advanced degree	57	24 (42.1%)	65	28 (43.1%)	
Annual Household Income, $50,000 and more	-	-	61	37 (60.7%)	-
Rurality, yes	-	-	60	31 (51.7%)	-
***Psychological Factors***					
MoCA (0–30)	58	26.2 ± 2.2	64	24.5 ± 3.0	***
Memory Complaint (0–14)	53	4.4 ± 2.2	64	5.5 ± 2.5	*
Depression (0–27)	56	2.4 ± 2.2	59	2.6 ± 2.7	
Anxiety (0–30)	55	3 ± 2.4	63	3.3 ± 2.9	
Perceived Stress (0–40)	57	9.4 ± 5.7	63	9.5 ± 5.8	
Emotion					
Negative Affect (10–50)	56	12.2 ± 3.6	-	-	-
Positive Affect (10–50)	56	35.6 ± 8.1	-	-	-
Cognitive Emotion Regulation					
Expressive Suppression (4–28)	-	-	56	13.1 ± 5.3	-
Cognitive Reappraisal (6–42)	-	-	54	32.9 ± 8.4	-
Self-Efficacy (10–40)	58	34.5 ± 3.9	63	35 ± 3.8	
Pervious Eating Disorder (0–5)	58	0.5 ± 0.8	64	0.7 ± 0.9	
Quality of Life (13–65)	57	59.8 ± 6.1	55	58.7 ± 5.7	
***Social and Behavioral Factors***					
Living Situation, with at least one other person	58	36 (62.1%)	65	37 (56.9%)	
Occupation, working	57	12 (21.1%)	64	16 (25.0%)	
Interpersonal Support (12–48)	55	39.9 ± 7.2	64	39.5 ± 6.3	
Physical Activity, Active	56	33 (58.9%)	64	24 (37.5%)	*
***Medical and Functional Factors***					
Functional Status					
Basic ADL (0–100)	48	96.1 ± 14.9	-	-	-
Intermediate ADL (0–100)	53	83.8 ± 26.9	-	-	-
Mental Health (0–100)	55	79.6 ± 16.6	-	-	-
Work Performance (0–100)	16	85.4 ± 20.4	-	-	-
Social Activities (0–100)	53	95.1 ± 15.7	-	-	-
Quality of Interactions (0–100)	57	87.4 ± 12.5	-	-	-
General Symptoms (0–120)	-	-	58	19.6 ± 15.0	-
Previous Weight Challenge, yes	40	23 (57.5%)	-	-	-
Pain (0–24)	58	3.9 ± 4.3	64	3.4 ± 4.6	
Sleep Quality (0–21)	51	5.9 ± 3.9	64	6.7 ± 3.5	
***Outcomes***					
Ketone Above Trace Level, % (0–100)	28	31.6 ± 29.3	28	3.2 ± 9.1	***
Perceived Adherence (0–10)	44	7.1 ± 1.9	56	6.2 ± 1.4	*
Mediterranean Diet Adherence (0–14)	-	-	52	7.9 ± 1.6	-
Session Attendance, % (0–100)	55	72.1 ± 28.9	65	84.5 ± 23.0	*
Submission of Food Log, yes	58	25 (43.1%)	65	55 (84.6%)	***

Note. Descriptive statistics are reported Mean ± SD or Count (Percentage). Group differences were evaluated by *t*-test (for continuous measures) and chi-square test (for categorical measures). *** *p* < 0.001, * *p* < 0.05.

## Data Availability

The data presented in this study is available upon request from the corresponding author due to ongoing analyses and the need to preserve the integrity of planned follow-up studies.

## Abbreviations

The following abbreviations are used in this manuscript:

SDOH	Social Determinants of Health
MED	Mediterranean diet
MMKD	Modified Mediterranean ketogenic diet
ICAN	Improving Cognitive Aging through Nutrition
MCI	Mild cognitive impairment

## Appendix A

**Table A1:** Correlations between the percentage of days with ketone levels above trace and each predictor.

	Pilot A						Pilot B					
Measures	Corr Method	Corr	Lower	Upper	*p*-Value	*p*-Value FDR	Corr Method	Corr	Lower	Upper	*p*-Value	*p*-Value FDR
**Sociodemographic Factors**												
Age	Spearman	−0.117	−0.534	0.292	0.553	0.845	Spearman	−0.116	−0.495	0.262	0.558	0.981
Sex	Spearman	−0.159	−0.526	0.200	0.418	0.837	Spearman	0.007	−0.383	0.414	0.974	0.981
Race	Spearman	−0.446	−0.698	−0.218	0.017	0.370	Spearman	0.056	−0.326	0.440	0.778	0.981
Education	Spearman	0.128	−0.238	0.501	0.525	0.845	Spearman	−0.005	−0.351	0.341	0.981	0.981
Income	NA	NA	NA	NA	NA	NA	Spearman	0.188	−0.166	0.547	0.367	0.981
Rurality	NA	NA	NA	NA	NA	NA	Spearman	0.025	−0.389	0.423	0.907	0.981
**Psychological Factors**												
MoCA	Spearman	0.414	0.082	0.784	0.028	0.370	Spearman	−0.376	−0.699	−0.072	0.049	0.581
Memory Complaint	Spearman	−0.071	−0.477	0.352	0.725	0.876	Spearman	0.108	−0.280	0.487	0.585	0.981
Depression	Spearman	−0.105	−0.565	0.334	0.603	0.870	Spearman	0.142	−0.231	0.503	0.481	0.981
Anxiety	Spearman	−0.134	−0.527	0.248	0.498	0.845	Spearman	0.075	−0.287	0.444	0.709	0.981
Stress	Spearman	0.093	−0.325	0.506	0.639	0.875	Spearman	−0.143	−0.521	0.229	0.478	0.981
Emotion												
Negative Affect	Spearman	0.127	−0.285	0.554	0.520	0.845	NA	NA	NA	NA	NA	NA
Positive Affect	Spearman	0.233	−0.167	0.622	0.242	0.698	NA	NA	NA	NA	NA	NA
Cognitive Emotion Regulation												
Suppression	NA	NA	NA	NA	NA	NA	Spearman	0.354	0.002	0.739	0.076	0.581
Positive Reappraisal	NA	NA	NA	NA	NA	NA	Spearman	0.042	−0.332	0.437	0.840	0.981
Self-Efficacy	Spearman	0.232	−0.141	0.622	0.234	0.698	Spearman	0.086	−0.281	0.472	0.675	0.981
Previous Eating Disorder	Spearman	−0.177	−0.555	0.176	0.369	0.837	Spearman	0.268	−0.115	0.647	0.169	0.969
Quality of Life	Spearman	0.270	−0.139	0.691	0.173	0.698	Spearman	−0.102	−0.510	0.302	0.621	0.981
**Social and Behavioral Factors**												
Living Situation	Spearman	0.256	−0.087	0.601	0.188	0.698	Spearman	−0.096	−0.499	0.273	0.626	0.981
Occupation Status	Spearman	0.058	−0.440	0.540	0.775	0.876	Spearman	0.014	−0.402	0.429	0.946	0.981
Interpersonal Support	Spearman	0.253	−0.158	0.694	0.203	0.698	Spearman	−0.049	−0.456	0.349	0.808	0.981
Physical Activity	Spearman	−0.010	−0.402	0.373	0.959	0.959	Spearman	0.183	−0.202	0.555	0.352	0.981
**Medical and Functional Factors**												
Functional Status												
Basic ADL	Spearman	0.399	0.179	0.596	0.059	0.514	NA	NA	NA	NA	NA	NA
Intermediate ADL	Spearman	0.045	−0.336	0.431	0.830	0.899	NA	NA	NA	NA	NA	NA
Mental Health	Spearman	−0.022	−0.407	0.374	0.913	0.950	NA	NA	NA	NA	NA	NA
Social Activities	Spearman	0.216	−0.070	0.495	0.299	0.778	NA	NA	NA	NA	NA	NA
Quality of Interactions	Spearman	−0.068	−0.450	0.321	0.737	0.876	NA	NA	NA	NA	NA	NA
General Symptoms	NA	NA	NA	NA	NA	NA	Spearman	0.107	−0.288	0.501	0.595	0.981
Previous Weight Challenge	Spearman	−0.070	−0.501	0.357	0.741	0.876	NA	NA	NA	NA	NA	NA
Pain	Spearman	−0.162	−0.515	0.180	0.410	0.837	Spearman	−0.124	−0.475	0.218	0.537	0.981
Sleep	Spearman	0.330	−0.072	0.756	0.124	0.698	Spearman	0.362	−0.007	0.750	0.058	0.581

Note. Corr = correlation, Lower = lower bound of the 95% bootstrap confidence interval, Upper = upper bound of the 95% bootstrap confidence interval, *p*-value FDR = *p*-value with false discovery rate control.

**Table A2:** Correlations between perceived adherence and each predictor.

	Pilot A						Pilot B					
Measures	Corr Method	Corr	Lower	Upper	*p*-Value	*p*-Value FDR	Corr Method	Corr	Lower	Upper	*p*-Value	*p*-Value FDR
**Sociodemographic Factors**												
Age	Spearman	−0.235	−0.517	0.023	0.124	0.404	Pearson	−0.038	−0.303	0.214	0.780	0.815
Sex	Spearman	−0.055	−0.338	0.227	0.724	0.818	Pearson	−0.139	−0.437	0.146	0.309	0.587
Race	Spearman	−0.382	−0.680	−0.096	0.010	0.157	Pearson	−0.161	−0.428	0.108	0.250	0.524
Education	Spearman	−0.268	−0.568	0.025	0.082	0.304	Spearman	0.077	−0.176	0.339	0.570	0.683
Income	NA	NA	NA	NA	NA	NA	Pearson	0.103	−0.156	0.370	0.461	0.663
Rurality	NA	NA	NA	NA	NA	NA	Pearson	−0.019	−0.290	0.251	0.895	0.895
**Psychological Factors**												
MoCA	Spearman	0.277	0.026	0.545	0.068	0.304	Spearman	0.118	−0.155	0.398	0.390	0.640
Memory Complaint	Spearman	−0.115	−0.438	0.222	0.479	0.732	Spearman	−0.163	−0.417	0.090	0.235	0.524
Depression	Spearman	−0.207	−0.503	0.078	0.184	0.435	Spearman	−0.363	−0.586	−0.153	0.008	0.063
Anxiety	Spearman	−0.231	−0.529	0.053	0.142	0.410	Spearman	−0.085	−0.313	0.144	0.543	0.683
Stress	Spearman	−0.150	−0.476	0.163	0.337	0.557	Spearman	−0.193	−0.482	0.060	0.162	0.524
Emotion												
Negative Affect	Spearman	0.150	−0.170	0.475	0.343	0.557	NA	NA	NA	NA	NA	NA
Positive Affect	Spearman	0.169	−0.160	0.499	0.277	0.515	NA	NA	NA	NA	NA	NA
Cognitive Emotion Regulation												
Suppression	NA	NA	NA	NA	NA	NA	Pearson	−0.040	−0.286	0.218	0.778	0.815
Positive Reappraisal	NA	NA	NA	NA	NA	NA	Spearman	0.112	−0.169	0.394	0.433	0.663
Self-Efficacy	Spearman	0.079	−0.222	0.385	0.611	0.818	Spearman	0.170	−0.090	0.441	0.218	0.524
Previous Eating Disorder	Spearman	−0.268	−0.557	0.017	0.079	0.304	Spearman	−0.133	−0.414	0.145	0.332	0.587
Quality of Life	Spearman	0.359	0.055	0.651	0.018	0.157	Spearman	0.268	0.013	0.518	0.055	0.253
**Social and Behavioral Factors**												
Living Situation	Spearman	0.208	−0.097	0.513	0.176	0.435	Pearson	0.362	0.138	0.587	0.006	0.063
Occupation Status	Spearman	0.064	−0.225	0.363	0.681	0.818	Pearson	−0.074	−0.350	0.191	0.594	0.683
Interpersonal Support	Spearman	0.293	−0.015	0.619	0.059	0.304	Spearman	0.170	−0.090	0.427	0.213	0.524
Physical Activity	Spearman	0.033	−0.302	0.361	0.831	0.831	Pearson	0.094	−0.180	0.364	0.492	0.666
**Medical and Functional Factors**												
Functional Status												
Basic ADL	Spearman	0.408	0.216	0.616	0.012	0.157	NA	NA	NA	NA	NA	NA
Intermediate ADL	Spearman	0.039	−0.301	0.410	0.809	0.831	NA	NA	NA	NA	NA	NA
Mental Health	Spearman	−0.062	−0.395	0.280	0.695	0.818	NA	NA	NA	NA	NA	NA
Social Activities	Spearman	0.179	−0.075	0.442	0.264	0.515	NA	NA	NA	NA	NA	NA
Quality of Interactions	Spearman	0.104	−0.211	0.440	0.507	0.732	NA	NA	NA	NA	NA	NA
General Symptoms	NA	NA	NA	NA	NA	NA	Spearman	−0.414	−0.635	−0.210	0.003	0.058
Previous Weight Challenge	Spearman	0.072	−0.268	0.404	0.671	0.818	NA	NA	NA	NA	NA	NA
Pain	Spearman	−0.170	−0.494	0.150	0.269	0.515	Spearman	−0.217	−0.464	0.028	0.111	0.426
Sleep	Spearman	0.048	−0.280	0.379	0.777	0.831	Spearman	−0.312	−0.575	−0.052	0.019	0.111

Note. Corr = correlation, Lower = lower bound of the 95% bootstrap confidence interval, Upper = upper bound of the 95% bootstrap confidence interval, *p*-value FDR = *p*-value with false discovery rate control.

**Table A3:** Correlations between Mediterranean diet adherence and each predictor (Pilot B only).

	Pilot B					
Measures	Corr Method	Corr	Lower	Upper	*p*-Value	*p*-Value FDR
**Sociodemographic Factors**						
Age	Pearson	0.099	−0.173	0.372	0.486	0.778
Sex	Pearson	−0.170	−0.519	0.153	0.228	0.476
Race	Pearson	−0.059	−0.357	0.220	0.686	0.930
Education	Spearman	0.141	−0.167	0.447	0.320	0.613
Income	Pearson	0.041	−0.263	0.341	0.780	0.930
Rurality	Pearson	−0.270	−0.526	−0.023	0.064	0.268
**Psychological Factors**						
MoCA	Spearman	0.019	−0.258	0.297	0.893	0.937
Memory Complaint	Spearman	−0.244	−0.523	0.017	0.082	0.268
Depression	Spearman	−0.286	−0.596	0.003	0.049	0.268
Anxiety	Spearman	−0.096	−0.367	0.183	0.508	0.778
Stress	Spearman	−0.046	−0.339	0.238	0.749	0.930
Emotion						
Negative Affect	NA	NA	NA	NA	NA	NA
Positive Affect	NA	NA	NA	NA	NA	NA
Cognitive Emotion Regulation						
Suppression	Pearson	−0.189	−0.443	0.066	0.188	0.433
Positive Reappraisal	Spearman	0.422	0.171	0.683	0.003	0.058
Self-Efficacy	Spearman	0.119	−0.190	0.432	0.410	0.725
Previous Eating Disorder	Spearman	−0.034	−0.294	0.235	0.809	0.930
Quality of Life	Spearman	0.251	−0.028	0.535	0.081	0.268
**Social and Behavioral Factors**						
Living Situation	Pearson	−0.045	−0.342	0.238	0.749	0.930
Occupation Status	Pearson	−0.019	−0.313	0.260	0.896	0.937
Interpersonal Support	Spearman	−0.008	−0.306	0.293	0.955	0.955
Physical Activity	Pearson	0.219	−0.032	0.482	0.120	0.344
**Medical and Functional Factors**						
Functional Status						
Basic ADL	NA	NA	NA	NA	NA	NA
Intermediate ADL	NA	NA	NA	NA	NA	NA
Mental Health	NA	NA	NA	NA	NA	NA
Social Activities	NA	NA	NA	NA	NA	NA
Quality of Interactions	NA	NA	NA	NA	NA	NA
General Symptoms	Spearman	−0.290	−0.562	−0.027	0.048	0.268
Previous Weight Challenge	NA	NA	NA	NA	NA	NA
Pain	Spearman	−0.274	−0.552	0.002	0.052	0.268
Sleep	Spearman	−0.196	−0.470	0.068	0.164	0.419

Note. Corr = correlation, Lower = lower bound of the 95% bootstrap confidence interval, Upper = upper bound of the 95% bootstrap confidence interval, *p*-value FDR = *p*-value with false discovery rate control.

**Table A4:** Correlations between session attendance and each predictor.

	Pilot A						Pilot B					
Measures	Corr Method	Corr	Lower	Upper	*p*-Value	*p*-Value FDR	Corr Method	Corr	Lower	Upper	*p*-Value	*p*-Value FDR
**Sociodemographic Factors**												
Age	Spearman	−0.052	−0.331	0.228	0.705	0.873	Spearman	−0.043	−0.280	0.206	0.733	0.977
Sex	Spearman	−0.135	−0.421	0.156	0.326	0.735	Spearman	−0.032	−0.269	0.203	0.800	0.977
Race	Spearman	−0.131	−0.377	0.117	0.339	0.735	Spearman	0.098	−0.157	0.360	0.448	0.977
Education	Spearman	0.155	−0.108	0.410	0.264	0.735	Spearman	0.194	−0.053	0.433	0.121	0.782
Income	NA	NA	NA	NA	NA	NA	Spearman	0.262	0.008	0.516	0.041	0.473
Rurality	NA	NA	NA	NA	NA	NA	Spearman	−0.021	−0.267	0.219	0.872	0.977
**Psychological Factors**												
MoCA	Spearman	0.139	−0.129	0.422	0.310	0.735	Spearman	0.328	0.081	0.575	0.008	0.189
Memory Complaint	Spearman	−0.169	−0.460	0.127	0.240	0.735	Spearman	0.105	−0.154	0.378	0.409	0.977
Depression	Spearman	−0.082	−0.368	0.196	0.558	0.846	Spearman	−0.004	−0.267	0.271	0.977	0.977
Anxiety	Spearman	0.289	0.038	0.544	0.037	0.641	Spearman	−0.085	−0.352	0.173	0.510	0.977
Stress	Spearman	0.242	0.010	0.484	0.078	0.641	Spearman	−0.007	−0.263	0.253	0.956	0.977
Emotion												
Negative Affect	Spearman	−0.070	−0.343	0.200	0.613	0.846	NA	NA	NA	NA	NA	NA
Positive Affect	Spearman	−0.030	−0.342	0.283	0.832	0.922	NA	NA	NA	NA	NA	NA
Cognitive Emotion Regulation												
Suppression	NA	NA	NA	NA	NA	NA	Spearman	−0.030	−0.300	0.241	0.828	0.977
Positive Reappraisal	NA	NA	NA	NA	NA	NA	Spearman	−0.189	−0.458	0.065	0.170	0.782
Self Efficacy	Spearman	−0.210	−0.452	0.022	0.123	0.641	Spearman	−0.075	−0.310	0.161	0.558	0.977
Previous Eating Disorder	Spearman	0.117	−0.160	0.389	0.397	0.794	Spearman	0.038	−0.205	0.284	0.768	0.977
Quality of Life	Spearman	−0.089	−0.352	0.173	0.523	0.846	Spearman	−0.100	−0.343	0.141	0.469	0.977
**Social and Behavioral Factors**												
Living Situation	Spearman	−0.018	−0.291	0.259	0.895	0.930	Spearman	0.135	−0.118	0.387	0.283	0.977
Occupation Status	Spearman	−0.099	−0.373	0.171	0.477	0.827	Spearman	−0.018	−0.287	0.257	0.885	0.977
Interpersonal Support	Spearman	−0.062	−0.354	0.223	0.660	0.858	Spearman	0.056	−0.201	0.314	0.659	0.977
Physical Activity	Spearman	−0.026	−0.301	0.257	0.851	0.922	Spearman	0.032	−0.215	0.278	0.800	0.977
**Medical and Functional Factors**												
Functional Status												
Basic ADL	Spearman	0.172	−0.131	0.481	0.258	0.735	NA	NA	NA	NA	NA	NA
Intermediate ADL	Spearman	0.228	−0.040	0.489	0.112	0.641	NA	NA	NA	NA	NA	NA
Mental Health	Spearman	−0.107	−0.379	0.157	0.444	0.824	NA	NA	NA	NA	NA	NA
Social Activities	Spearman	−0.203	−0.528	0.112	0.157	0.679	NA	NA	NA	NA	NA	NA
Quality of Interactions	Spearman	−0.031	−0.273	0.209	0.824	0.922	NA	NA	NA	NA	NA	NA
General Symptoms	NA	NA	NA	NA	NA	NA	Spearman	−0.009	−0.273	0.247	0.946	0.977
Previous Weight Challenge	Spearman	0.313	0.033	0.588	0.052	0.641	NA	NA	NA	NA	NA	NA
Pain	Spearman	−0.069	−0.350	0.208	0.618	0.846	Spearman	0.179	−0.043	0.397	0.156	0.782
Sleep	Spearman	0.009	−0.297	0.307	0.953	0.953	Spearman	0.053	−0.193	0.297	0.678	0.977

Note. Corr = correlation, Lower = lower bound of the 95% bootstrap confidence interval, Upper = upper bound of the 95% bootstrap confidence interval, *p*-value FDR = *p*-value with false discovery rate control.

**Table A5:** Correlations between food log submissions and each predictor.

	Pilot A						Pilot B					
Measures	Corr Method	Corr	Lower	Upper	*p*-Value	*p*-Value FDR	Corr Method	Corr	Lower	Upper	*p*-Value	*p*-Value FDR
**Sociodemographic Factors**												
Age	Pearson	−0.110	−0.365	0.128	0.412	0.847	Pearson	0.006	−0.277	0.301	0.959	0.959
Sex	phi	−0.155	−0.391	0.079	0.246	0.779	phi	0.046	−0.190	0.277	0.718	0.959
Race	phi	−0.154	−0.386	0.081	0.254	0.779	phi	−0.111	−0.345	0.122	0.390	0.959
Education	Spearman	0.198	−0.060	0.455	0.141	0.732	Spearman	0.258	0.025	0.498	0.038	0.349
Income	NA	NA	NA	NA	NA	NA	phi	0.278	0.038	0.521	0.030	0.349
Rurality	NA	NA	NA	NA	NA	NA	phi	−0.164	−0.408	0.069	0.210	0.959
**Psychological Factors**												
MoCA	Spearman	0.211	−0.039	0.461	0.112	0.732	Spearman	0.114	−0.162	0.392	0.371	0.959
Memory Complaint	Spearman	−0.257	−0.501	−0.006	0.063	0.732	Spearman	0.029	−0.211	0.273	0.817	0.959
Depression	Spearman	0.078	−0.182	0.356	0.566	0.847	Spearman	−0.086	−0.375	0.217	0.519	0.959
Anxiety	Spearman	0.121	−0.153	0.376	0.379	0.847	Spearman	−0.052	−0.323	0.211	0.686	0.959
Stress	Spearman	−0.051	−0.326	0.222	0.708	0.847	Spearman	0.028	−0.260	0.304	0.831	0.959
Emotion												
Negative Affect	Spearman	−0.150	−0.411	0.115	0.270	0.779	NA	NA	NA	NA	NA	NA
Positive Affect	Pearson	0.106	−0.161	0.377	0.435	0.847	NA	NA	NA	NA	NA	NA
Cognitive Emotion Regulation												
Suppression	NA	NA	NA	NA	NA	NA	Pearson	0.018	−0.349	0.357	0.894	0.959
Positive Reappraisal	NA	NA	NA	NA	NA	NA	Spearman	−0.273	−0.543	−0.006	0.046	0.349
Self Efficacy	Spearman	0.007	−0.247	0.270	0.956	0.956	Spearman	−0.012	−0.253	0.231	0.926	0.959
Previous Eating Disorder	Spearman	0.054	−0.211	0.316	0.688	0.847	Spearman	0.195	−0.017	0.408	0.123	0.705
Quality of Life	Spearman	−0.012	−0.258	0.241	0.929	0.956	Spearman	0.055	−0.156	0.270	0.688	0.959
**Social and Behavioral Factors**												
Living Situation	phi	0.035	−0.222	0.284	0.796	0.900	phi	−0.026	−0.270	0.225	0.834	0.959
Occupation Status	phi	−0.110	−0.361	0.143	0.417	0.847	phi	−0.050	−0.330	0.218	0.697	0.959
Interpersonal Support	Spearman	0.012	−0.259	0.279	0.933	0.956	Spearman	0.062	−0.209	0.333	0.627	0.959
Physical Activity	phi	0.093	−0.162	0.348	0.497	0.847	phi	0.067	−0.181	0.308	0.601	0.959
**Medical and Functional Factors**												
Functional Status												
Basic ADL	Spearman	−0.054	−0.333	0.244	0.716	0.847	NA	NA	NA	NA	NA	NA
Intermediate ADL	Spearman	0.191	−0.065	0.462	0.170	0.737	NA	NA	NA	NA	NA	NA
Mental Health	Spearman	−0.204	−0.466	0.059	0.135	0.732	NA	NA	NA	NA	NA	NA
Social Activities	Spearman	−0.084	−0.369	0.191	0.550	0.847	NA	NA	NA	NA	NA	NA
Quality of Interactions	Spearman	−0.063	−0.341	0.213	0.640	0.847	NA	NA	NA	NA	NA	NA
General Symptoms	NA	NA	NA	NA	NA	NA	Spearman	−0.047	−0.308	0.223	0.726	0.959
Previous Weight Challenge	phi	0.311	0.008	0.607	0.050	0.732	NA	NA	NA	NA	NA	NA
Pain	Spearman	−0.065	−0.317	0.177	0.625	0.847	Spearman	0.096	−0.134	0.348	0.449	0.959
Sleep	Spearman	0.101	−0.154	0.375	0.482	0.847	Spearman	0.023	−0.194	0.254	0.854	0.959

Note. Corr = correlation, Lower = lower bound of the 95% bootstrap confidence interval, Upper = upper bound of the 95% bootstrap confidence interval, *p*-value FDR = *p*-value with false discovery rate control.
